# Benefits of an early cooling phase in continuous renal replacement therapy for ICU patients

**DOI:** 10.1186/2110-5820-2-40

**Published:** 2012-08-23

**Authors:** René Robert, Jean-Eudes Méhaud, Najette Timricht, Véronique Goudet, Olivier Mimoz, Bertrand Debaene

**Affiliations:** 1 Department of Critical Care Medicine, University of Poitiers, CHU, Poitiers, F86000, France; 2 INSERM Unit U1082, University of Poitiers, CHU, Poitiers, F86000, France; 3 Department of Anesthesiology, Centre Hospitalier de Pau, Pau, F64000, France; 4 INSERM ERI 23, University of Poitiers, CHU, Poitiers, F86000, France; 5 Service de Réanimation Médicale, CHU Poitiers, Hôpital Jean Bernard, Poitiers Cedex, 86021, France

**Keywords:** Renal replacement therapy, Hemofiltration, Hemodynamic, Rewarming device, Temperature

## Abstract

**Background:**

Lowering the temperature setting in the heating device during continuous venovenous hemofiltration (CVVH) is an option. The purpose of this study was to determine the effects on body temperature and hemodynamic tolerance of two different temperature settings in the warming device in patients treated with CVVH.

**Methods:**

Thirty patients (mean age: 66.5 years; mean SAPS 2: 55) were enrolled in a prospective crossover randomized study. After a baseline of 2 h at 38°C, the heating device was randomly set to 38°C (group A) and 36°C (group B) for 6 h. Then, the temperatures were switched to 36°C in group A and to 38°C in group B for another 6 h. Hemodynamic parameters and therapeutic interventions to control the hemodynamics were recorded.

**Results:**

There was no significant change in body temperature in either group. During the first 6 h, group B patients showed significantly increased arterial pressure (*p* = 0.01) while the dosage of catecholamine was significantly decreased (*p* = 0.04). The number of patients who required fluid infusion or increase in catecholamine dosage was similar. During the second period of the study, hemodynamic parameters were unchanged in both groups.

**Conclusions:**

In patients undergoing CVVH, warming of the substitute over 36°C had no impact on body temperature. We showed that setting the fluid temperature at 36°C for a period of time early in the procedure is beneficial in terms of increased mean arterial pressure and decreased catecholamine infusion dosage.

## Background

The thermal consequence of renal replacement therapies varies with the technique used. During IHD, core temperature (CT) increase is due to: 1)the direct delivery of thermal energy by the extracorporeal circuit that overcomes the external energy losses; 2) the increase in thermogenic metabolic rate as a consequence of biological mechanisms induced by the bioincompatibility of the circuit materials; and 3) decreased dissipation of heat caused by cutaneous vasoconstriction compensating for ultrafiltration-related hypovolemia [1-4]. As summarized in a meta-analysis [5], several studies demonstrated the beneficial effect of dialysate cooling on vascular response, with a reduction of intradialytic hypotensive episodes and higher postdialysis mean arterial pressure. As a consequence, it is now recommended to decrease the temperature of the dialysate to improve hemodynamic tolerance of IHD [6].

In intensive care unit (ICU) patients with multiple organ failure, continuous renal replacement therapies (CRRT) are frequently used. During CRRT, the consequence of heat loss in ECC is a reduced body temperature, which in turn may activate compensatory mechanisms, such as skin vasoconstriction and endogenous heat production [7]. Hypothermia can theoretically induce detrimental effects [8,9], such as uncontrolled, intense shivering, uncoordinated movements, pain and discomfort, decreased insulin secretion and peripheral sensitivity, and left shift in the oxyhemoglobin dissociation curve, which may impair O_2_ extraction rate. Thus, manufacturers included in the hemofiltration machine a heating device to warm the blood returning to the patient aiming at counteracting the heat loss, and current practice for CRRT is to warm up substitution fluids to avoid significant hypothermia [10]. However, such warming may alter vascular reactivity and induce hypotension [11]. The optimal target temperature remains unknown, and there are no guidelines for the setting of the heating device during hemofiltration, particularly in septic patients.

In patients with chronic kidney injury receiving hemofiltration, it has been reported that cooling the circuit may increase vascular resistances and improve hemodynamic tolerance [12,13]. However, few studies have analyzed the temperature variations in ICU patients treated with continuous renal therapies. Manthous et al. [14] reported major hypothermia associated with shivering and vasoconstriction in a short series of septic patients. Other studies failed to demonstrate any deleterious hemodynamic effect of hypothermia in patients treated with CVVH [15,16]. More recently, Rokyta et al. demonstrated in patients treated with CVVH that cooling over a short period of time was associated with significant decrease in heart rate, cardiac output, systemic oxygen delivery and consumption, as well as increase of the mean arterial pressure without significant alteration of energetic balance [17]. Thus, further investigation is of importance to determine the optimal recommended temperature in ICU patients treated with CVVH.

The goal of this study was to examine the eventual benefits of temperature changes in the fluid substitute on hemodynamic tolerance in ICU patients treated with CVVH.

## Methods

### *In vitro* validation study

Because it was not possible to monitor the real temperature variation in the circuit induced by the different levels of heating device setting, in a preliminary phase, the hemofiltration circuit was set *in vitro*, using CRRT machine a (Multifiltrate generator, Fresenius medical Care; Bad Homburg, Germany) and polysulfone membrane (Ultraflux AV1000S, Fresenius medical Care; Bad Homburg, Germany). Access and return lines were connected to a 2,000-ml saline bag placed on a heating device constantly set at 39°C. Blood flow was set to 100 ml per min and ultrafiltration (UF) rate (2000 ml per h) was continuously compensated with saline at the same rate. A thermistor sensor (Hanna Instruments; NJ, USA) was inserted inside the bubble trap close to the tip of venous line. Temperature of the heating device incorporated to the Multifiltrate generator was first set to 36°C then to 38°C and back to 36°C.

Two experiments were successively performed. After 30 min of stability, the measured temperature was 32°C in both cases while the heating device was set to 36°C. After setting the heating device to 38°C, and in the subsequent 30 min, the temperature increased to 32.8°C in the first experiment and 32.7°C in the second. Turning back the heating device to 36°C, the measured temperature decreased to 32°C in both experiments. Thus, moderate, but significant, temperature variations were induced using two different levels of temperature setting in the warming device suggesting that true blood temperature differences may be achieved *in vivo* with similar heating device setting

### Patient study

#### Patient recruitment

Medical or surgical ICU patients were recruited for this monocenter pilot crossover randomized study.

#### Sample size

In the absence of data from literature allowing determination of the frequency of hypotension during the first 6 h of hemofiltration, this study was a pilot study involving 30 patients (15 in each group). The duration of the study was 12 h.

#### Inclusion and exclusion criteria

Patients with acute renal failure and multiple organ failure treated with CVVH for at least 6 h were included. The weight loss was to be lower than 50 ml per hour to be included in the study. The choice of CVVH as replacement technique was determined by the physician in charge of the patient. Hemodynamic failure was defined by the necessity to administer catecholamine infusion after optimal fluid infusion (norepinephrine > 0.1 μg/kg/min; epinephrine > 0.1 μg/kg/min or dobutamine > 10 μg/kg/min) to achieve a mean arterial pressure higher than 65 mmHg. Patients admitted after cardiac resuscitation and patients with a high bleeding risk without possibility to use heparin as anticoagulation in the circuit were not included in the study. Other patient exclusion criteria were: existing treatment with hemodiafiltration (CVVHDF); major hemodynamic instability defined by the necessity of increased catecholamine infusion dosage or to infuse fluid challenge to control arterial pressure within the first 2 hours following the start of CVVH; tympanic temperature <36°C before randomization.

#### Consent - ethics

Institutional approval was obtained from the Ethical Committee (Comité de Protection des Personnes CHU Poitiers, 86021 Poitiers Cedex, France; Chairperson Dr Louis Lacoste; N°2007.A00681-52). Written, informed consent was obtained from the patients or next of kin.

#### Randomization procedure

The order of the sessions was randomized by using a randomization list.

#### Instruments - machines used

CVVHF was set up following a standardized institutional protocol. Briefly, a double-lumen, 13-French catheter was positioned in the femoral (20 or 25 cm of length) or internal jugular vein (20 cm). UF rate was set to 35 ml/kg/h with a corresponding adapted blood flow between 150 and 200 ml/min. Fluid replacement was administered before (1/3) and after dilution (2/3). Multifiltrate generator (Fresenius Medical Care; Bad Homburg, Germany) and polysulfone membrane (Ultraflux AV1000S, Fresenius Medical Care) were used. Anticoagulation was performed with unfractionated heparin targeting activated coagulation time around 1.5 compared with control. The temperature of the heating device incorporated to the Multifiltrate generator and placed on the pre and post-dilution substrate line was initially set to 38°C in all patients.

#### Method - procedures

**Figure 1:**
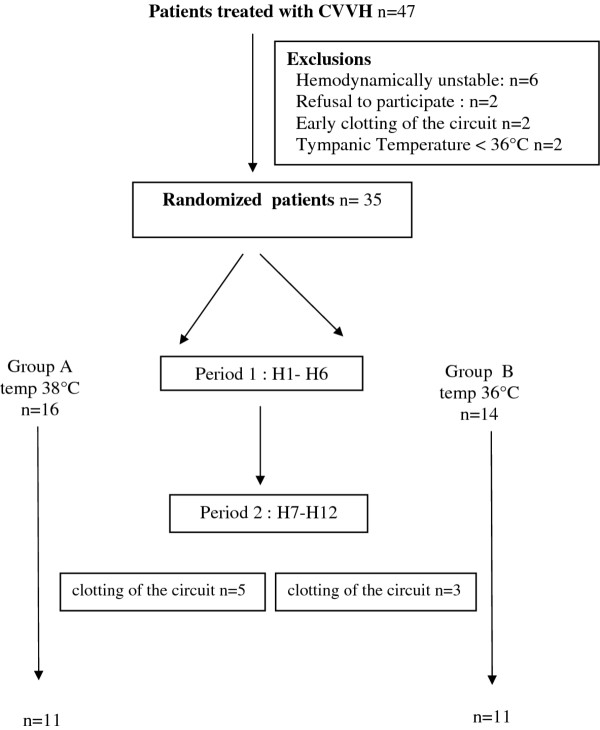
**Flow chart of the study. **Temp = temperature level set in the heating device of hemofiltration.

A schematic of the study is displayed in Figure 1.Two hours following the start of CVVH (baseline), the patients were randomized in two groups: in group A, the heater was set to 38°C during 6 h (H1-H6) and then to 36° for the next 6 h (H7-H12); conversely, in group B the heater was set initially to 36°C and then to 38° for similar periods of time.

#### Data collected

Tympanic temperature (Genius 2; Codivien-Nelcor and Puritan Benett; Boulder, CO), arterial pressure, and heart rate were monitored hourly from H1 to H12. Lactate levels and blood gas analysis were determined at H0, H6, and H12. The main judgement criteria were the occurrence of hemodynamic alteration during the considered 6 h period of time, defined as follows: decrease of mean arterial pressure (MAP) > 20% from initial value or the necessity of any therapeutic intervention, fluid infusion, or increase of catecholamine infusion dosage.

After the 12^th^ hour, the temperature setting left at the liberty of the physician in charge of the patient. If the lifespan of the circuit was below 6 h, the patient was excluded from the study. If the lifespan of the circuit was between 6 and 12 h, patient data were included in a secondary analysis.

### Statistical analysis

Qualitative data were compared using chi-squared test or Fisher exact test when appropriate. Continuous data were compared between groups using nonparametric Mann-Whitey test. Intragroup comparisons of continuous data were performed using a nonparametric Wilcoxon test for repeated measures. A *p* value < 0.05 was considered significant.

## Results

Among 47 patients treated with CVVH in our unit from August 2008 to may 2009, 17 were not included in the study (6 were too hemodynamically instable, 2 refused to participate, 7 had CVVH circuit dysfunction or clotting before H6 (2 before randomization and 5 early after randomization), and 2 had tympanic temperature below 36°C before randomization). The remaining 30 patients (21 males and 9 females) were included in the study (Figure 1). The clinical characteristics of these patients are summarized in Table 1.

**Table 1:** Clinical characteristics of the patients included in the study

	**Total n = 30**	**Group A n = 16**	**Group B n = 14**
Age (yr)	66.5 [10.3]	68.3 [12.3]	65.2 [7.3]
Sex male (no.patients)	21	12	9
SAPS 2 on admission	55.0 [14.1]	60.5 [12.0]	50.0 [13.9]
SOFA on admission	10.6 [4.6]	12.8 [3.8]	8.0 [3.8]
Main diagnosis (no. patients)			
Severe sepsis	20	12	8
Abdominal	7	6	1
Pneumonia	6	2	4
Miscellaneous	7	4	3
Cardiac surgery	4	1	3
Respiratory failure	2	1	1
Mesenteric ischemia	2	1	1
Temperature (°C) at ICU admission	37.4 [1.2]	37.4 [1.1]	37.4 [1.4]
SAP mmHg H1	117 [30]	118 [26]	113 [26]
DAP mmHg H1	58 [11]	58 [13]	58 [9]
MAP mmHg H1	75 [12.5]	77 [15]	71 [7]
Heart rate (beat/min) H1	98 [19]	94 [16]	103 [23]
Mechanical ventilation (n of patients)	27	16	11
Creatinine before onset (μmol/L)	267 [129]	257 [84]	293 [164]
Diuresis < 100 ml/6 h before inclusion (no. patients)	20	12	8
Hydrocortisone treatment (no. patients)	17	9	8
Norepinephrine (no. patients)	23	12	11
Dobutamine (no. patients) > 5 μg/kg/h	5	2	3
Epinephrine (no. patients)	3	2	1
Lactate (mmol/L)	3.7 [3.6]	4.1 [3.4]	3.2 [2.3]

Values are expressed as mean [SD] or median (interquartile range); *SAP* systolic arterial pressure, *DAP* diastolic arterial pressure, *MAP* mean arterial pressure H0 time of start of hemofiltration, *SAPS* simplified acute physiologic score SOFA score of organ failure.

The mean tympanic temperature before the start of CVVH was 37.2 ± 1.4°C in group A and 37.94 ± 1.1°C in group B. During the 2 hours of stabilization, the temperatures significantly decreased to 36.5 ± 0.7°C and 36.5 ± 0.9°C respectively. The temperature then remained unchanged for the duration of the study: 36.3 ± 0.5°C in group A and 36.4 ± 0.5°C in group B at H6; then 36.7 ± 1.1°C in group A and 36.1 ± 0.7°C in group B at H12.

During the first 6 h, group B patients significantly increased their arterial pressure (*p* = 0.01), whereas in these patients the dosage of catecholamine infusion was significantly decreased (*p* = 0.04; Table 2). Conversely in group A patients, there was no significant change in arterial pressure or catecholamine infusion dosage (Table 2). The number of patients requiring fluid infusion or increased catecholamine dosage was similar between groups (Table 2).

**Table 2:** Hemodynamic variations and therapeutic interventions from H1 to H6

	**Group A**				**Group B**				**P intergroup**
	**Period 1 temperature 38°C**				**Period 1 temperature 36°C**				
	**n = 16**				**n = 14**				
	**H1**	**H6**	**H6-H1**	**P Intra- group**	**H1**	**H6**	**H6-H1**	**P Intra-group**	**H6-H1**
SAP mmHg	118 [26]	118 [26]	0.2 [18.9]	0.73	113 [26]	125 [20]	12.3[4.4]	0. 01	0.06
DAP mmHg	58 [13]	60 [11]	1.4 [9]	0.65	58 [9]	65 [10]	6.3 [10.6]	0.055	0.28
MAP mmHg	77 [15]	78 14]	1.2 [11.4]	0.67	71 [7]	80 [12]	8.9 [11]	0.01	0.08
HR beat/min	95 [16]	92 [18]	−3 [5.9]	0.06	103 [23]	97 [28]	−5.5 [28.3]	0.49	0.86
Lactates (mmol/L)	4.1 [3.4]	4.9 [4.5]	0.4 [2.2]	0.58	3.2 [2.3]	2.6 [2.0]	0.1 [0.3]	0.31	0.2
Nep dosage μg/kg/min	0.47 [1.19]	0.47 [1.33]	0.013 [0.2]	0.72	0.93 [0.81]	0.62 [0.56]	−0.55 [0.3]	0.04	0.15
Increase of CA dose (no. patients)	6				4				
Decrease of CA dose (no. patients)	6				7				
Fluid infusion (no. patients)	5				2				
Total volume infused (ml)	3500				2500				
Therapeutic action (no. patients)	8				5				

Values are expressed as mean [SD] or median (interquartile range); *SAP* systolic arterial pressure, *DAP* diastolic arterial pressure, *MAP* mean arterial pressure, *Nep* norepinephrine, *CA* catecholamine. Therapeutic intervention: fluid infusion or increase dosage of catecholamine. P intragroup: H6 vs. H1.

Among the 30 patients, filter thrombosis occurred in 8 cases between H7 and H12 (5 patients from group A and 3 from group B), thus only 22 patients could complete the crossover study. The characteristics of the patients during the second period of time are summarized in Table 3. There was no difference observed between H7 and H12 in hemodynamic parameters, including lactate plasma level, or in therapeutic interventions.

**Table 3:** Hemodynamic variations and therapeutic interventions from H7 to H12

	**Group A**				**Group B**				**Pintergroup**
	**Period 2 Temp 36°C**				**Period 2 Temp 38°C**				
	**n = 11**				**n = 11**				
	**H7**	**H12**	**H12-H6**	**P Intra- group**	**H7**	**H12**	**H12-H7**	**P Intra- group**	**H12-H7**
SAP mmHg	124 [21]	125 [18]	1.1 [21.1]	0.66	121 [27]	122 [25]	1.5[12.8]	0.48	0.07
DAP mmHg	62 [12]	58 [10]	−3.5 [10.7]	0.45	60 [5]	64 [10]	3.7 [10]	0.18	0.16
MAP mmHg	79 [15]	79 [11]	−0.9 [12.3]	0.88	80 [10]	83 [13]	2.5 [9.9]	0.33	0.53
HR beat/min	88 [16]	89 [12]	1.4 [12.3]	0.99	105 [23]	102 [23]	−2.8 [6.4]	0.2	0.69
Lactates (mmol/L)	4.9 [5.7]	5.6 [6.8]	0.6 [1.4]	0.33	2.2 [1.6]	2.4 [1.9]	0.2 [0.3]	0.21	0.64
Nep dosage μg/kg/min	0.76 [1.01]	0.81 [1.13]	0.05 [0.3]	0.89	0.64 [0.58]	0.66 [0.63]	−0.02 [0.1]	0.67	0.99
Increase of CA dose (no. patients)	2				4				
Decrease of CA dose (no. patients)	4				2				
Fluid infusion (n of patients)	3				0				
Total volume infused (ml)	3500				0				
Therapeutic action (no. patients)	3				4				

Values are expressed as mean [SD] or median (interquartile range); *SAP* systolic arterial pressure, *DAP* diastolic arterial pressure, *MAP* mean arterial pressure, *CA* catecholamine, *Nep* norepinephrine. Therapeutic action: fluid infusion or increase dosage of catecholamine.

## Discussion and conclusions

Only a few studies have systematically investigated temperature changes during CRRT and evaluated their relationship to hemodynamics in ICU patients [14-17]. In one of these studies, marked hypothermia induced by CRRT led in some patients to shivering, profound peripheral vasoconstriction, and immune system dysfunction [14]. Yagi et al. reported that patients experienced temperature <35.5°C in 31 of the 67 of the CVVH sessions without deleterious effect of hypothermia [16]. In this latter study, there was no heating device within the circuit. In our study, the tympanic temperature rapidly decreased in both groups after the start of hemofiltration. Only two patients had temperature <35.5°C and were excluded from the study before randomization, as specified in the protocol, and no patient had temperature <35.5°C during the study period of the protocol. There were no tympanic differences for either temperature setting in the heating device (36°C or 38°C). However, because the heating device was placed on the infusate line, persisting energy transfer on the remaining part of the tubing may have attenuated the effect of the warming. We did not measure energy transfer in the circuit directly, but our *in vitro* experiment demonstrated a potential difference of 0.7°C to 0.8°C close to the venous return site of the circuit. This decrease was probably too low to induce significant differences in tympanic temperature of the patients. In the study byRokyta et al. despite induced high variations in temperature setting, only moderate change in cutaneous temp (0.9°C) and limited CT (1.3°C) were observed [17].

In chronic renal failure patients, several studies reported a significant reduction in the frequency of hypotensive episodes during hemofiltration [11-13], but in ICU patients the impact of temperature variation induced by CVVH on hemodynamic parameters is not established. Matamis et al. reported an increase of MAP and SVR only in patients who became hypothermic during hemofiltration [15]. Conversely, in one retrospective study, MAP was similar in hypothermic and nonhypothermic patients [16]. Finally, in a study involving nine patients submitted to a dramatic cooling of the circuit from 39°C to 20°C, there was a significant increase of vascular resistance and mean arterial pressure, whereas heart rate, cardiac output, systemic oxygen delivery, and consumption decreased [17]. However, in this study, hypothermia lasted only 2 hours, and potential deleterious effect could be observed.

Our study is the first prospective, controlled study to show a significant increase of SAP and MAP in a group of patients treated with CVVH set at a low temperature (36°C) for 6 h after a baseline period of 2 h at 38°C. This increase was associated with a significant decrease in catecholamine infusion dosage, indicating improvement of hemodynamic status. The suspected mechanisms for cardiovascular tolerance during cold HD are a greater catecholamine release, an increase in vascular peripheral resistances, and in venous tone [18]. In our practice, noninvasive investigation are privileged to manage hemodynamic instability and invasive monitoring was not systematically performed in our patients, thus the increase of vascular resistances during the 36°C period could not be demonstrated. Interestingly and contrasting with other studies, improvement of hemodynamic condition was not associated with differences in core temperature. This is in accordance with a study performed in chronic patients, showing that postdilution hemodiafiltration with a 2.5 l.h^-1^ volume exchange was associated with a better blood pressure stability compared with hemodiafiltration with low-volume exchange of dialysis without cooling, whereas CT was not decreased [4]. This was explained by the increased loss of extracorporeal energy, which may prevent cutaneous vasodilation [11].

There was no significant hemodynamic difference during the second period of the crossover experiment. On the one hand, maintenance of elevated SAP and MAP as well as reduced catecholamine infusion dosage suggest that this short period of hypothermia induces long-term benefits for the patient without the need for prolonged hypothermia and associated risks. On the other hand, the absence of hemodynamic difference during the second period of the crossover experiment in group A (lowered to 36°C at H6) suggests that the beneficial hemodynamic effects of the 36°C compared with the 38°C temperature setting is not straightforward. Timing the hypothermic period early in the treatment seems to be beneficial compared with a later hypothermic period. However, specific effect of temperature variation during a prolonged observation period may be blunted by many factors that may interfere with the hemodynamic status of a critically ill patient. Additionally, the reduced number of patients of the second period due to hemofilter dysfunction has probably limited the statistical power of the study.

Furthermore, the beneficial effect of cooling on the life span of the extracorporeal circuit has been suggested [19]. In our series, the relatively short duration of the temperature variation period and the limited number of patients did not allow evaluation of the impact of modest hypothermic temperature on the potential reduction in extracorporeal clotting and we did not evaluate the consequences of the two regimen of temperature on blood coagulation tests.

Our study has several limitations: 1) tympanic temperature measure may not precisely indicate CT, and the absence of body temperature difference might be related to the lack of accuracy of the technique [20]. However, there was no intraindividual variation of temperature for the duration of the study; 2) the duration of the study periods may have been too short for the evaluation of the hemodynamic consequences of these two setting regimens; 3) better temperature setting for the heating device (e.g., 39°C vs. 36°C) might have been more efficient to evidence hemodynamic differences. However, we chose these levels to be comparable with the studies performed during IHD, even though the thermal energy involved mechanisms are different, as we indicated; 4)finally, only a limited number of patients were enrolled in this study. However, this number was similar or higher than in most of the studies performed on the effect of temperature variation in ICU patients undergoing CRRT.

In conclusion, our study suggests that warming the infusate over 36°C has no impact on body temperature and may not be indicated in critically ill patients undergoing CVVH to limit heat loss. We show that setting the fluid temperature at 36°C in CVVH for a short period of time at the beginning of the treatment increased mean arterial pressure while allowing for a decrease in catecholamine infusion dosage. We believe this pilot study warrants an investigation involving a larger number of patients to confirm these findings.

## Competing interest

The authors declare that they have no competing interests.

## Authors’ contribution

RR and JEM conceived of the study and participated in its design and coordination. RR and JEM performed the associated experimental study. RR, JEM, NT, and VG were responsible for inclusion of the patients and data collection. RR, JEM, NT, and VGperformed the statistical analysis and drafted the manuscript. OM and BG helped with the conception of the study and reviewed the manuscript. RR, JEM, and NT: conception of the study, interpretation of data. JEM, NT, VG: in charge of inclusions, data management. RR, JEM: drafting the manuscript. RR, OM, BD: revision of the manuscript. All authors read and approved the final manuscript.
